# Accelerated Cognitive Function Decline in Community-Dwelling Older Adults during COVID-19 Pandemic: The Korean Frailty and Aging Cohort Study (KFACS)

**DOI:** 10.3390/ijerph191710666

**Published:** 2022-08-26

**Authors:** Jaehoon Jung, Sunyoung Kim, Byungsung Kim, Miji Kim, Jisoo Yang, Dongmin Chung, Changwon Won

**Affiliations:** 1 Department of Family Medicine, Kyung Hee University Medical Center, Seoul 02477, Korea; 2 Department of Family Medicine, College of Medicine, Kyung Hee University, Seoul 02477, Korea; 3 Department of Biomedical Science and Technology, College of Medicine/East-West Medical Research Institute, Kyung Hee University, Seoul 02477, Korea; 4 Elderly Frailty Research Center, Department of Family Medicine, College of Medicine, Kyung Hee University, Seoul 02477, Korea

**Keywords:** aged, COVID-19, cognition, Korea

## Abstract

This study aimed to analyze the effect of the COVID-19 pandemic on cognitive function of community-dwelling elderly individuals. Five-year (2016 to 2020) longitudinal data of the Korea Frailty and Aging Cohort Study (KFACS) were used. There were 1559 participants in 2016 and 1455 in 2017 aged 72–84 years. Follow-up was conducted at two-year intervals. We selected participants from the database of the 2017 and 2018 surveys for intergroup comparison over 2-year follow-ups. The number of study patients in the 2017-Group was 1027 and that of the 2018-Group was 879. In the intergroup comparison, the mean difference of word list memory score from 2018 to 2020 was −0.14, while that from 2017 to 2019 was 0.53. The mean difference of word list recall score from 2018 to 2020 was −0.25, while that from 2017 to 2019 was 0.03. These were significant even after adjusting confounding variables. In the intragroup comparison, the word list memory and recall scores from 2018 to 2020 were more decreased than those from 2016 to 2018. Conclusively, cognitive function of the Korean elderly cohort declined much more during the COVID-19 pandemic than before the pandemic, particularly in terms of memory and recall function.

## 1. Introduction

The coronavirus SARS-CoV2 (Severe Acute Respiratory Syndrome Coronavirus 2, COVID-19) was first identified in December 2019 [1] and then quickly spread all over the world, leading to the World Health Organization declaring it as a pandemic on 11 March 2020. Since the COVID-19 pandemic began in 2020, there have been many health problems and most countries have implemented strong social distancing policies to control the spread of the virus. To control the COVID-19 infection, the South Korean government implemented social distancing policies on 29 February 2020 [2]. During the pandemic, the elderly, who were at high risk of COVID-19 infection, were mandated to stay away from their families and communities to avoid infection. The elderly engaged in less outdoor activities compared to the younger population [3]. It has been shown that engaging in social activities stimulates the sensory system, self-esteem, affectivity, and emotional and psychological functions in elderly people [4]. The decreased social relationship and interaction during the COVID-19 pandemic could have profound psychological impact on the elderly [5]. Although some studies have shown cognitive decline in COVID-19 patients [6,7] or accelerated cognitive decline in patients with dementia and mild cognitive impairment [8]. A relatively high frequency of cognitive impairments in executive functioning, processing speed, category fluency, memory encoding, and recall were predominant among hospitalized patients (mean age 49 years) several months after patients contracted COVID-19 [9]. The angiotensin-converting enzyme 2 receptor was found in high concentrations in the mouse in substantia nigra, ventricles, middle temporal gyrus, posterior cingulate cortex, and olfactory bulb, as well as in the motor cortex and brain stem, and this distribution may explain some neurological complications in patients with COVID-19 [10]. A report says synergistic effects of systemic virus-mediated inflammation and transient hypoxia yield a dysfunction of the fronto-insular cortex, a signature of CNS involvement in neuro-COVID-19 [11].

However, there are only a few studies on the indirect effect of the COVID-19 pandemic on decline in cognitive functions in community-dwelling older adults. Cognitive function refers to a variety of mental abilities such as perception, attention, concentration, memory, decision-making, executive functions, motor skills and construction, and processing speed [12].

A Japanese cohort study showed that social isolation during the COVID-19 pandemic was associated with a decline in subjective cognitive function among older adults [13]. However, this study only used a subjective questionnaire on cognitive function and there was no comparative group. A French cohort which included participants aged 80 years or older showed an acceleration of cognitive decline after the start of the pandemic compared to the slow decline of the cognitive score observed during the 15 years preceding the pandemic [14]. However, this study used telephone interviews for cognitive status and no adjustment was made for confounders such as social activities, physical activities, and depression. Therefore, a comparative study that examines the decline in objective cognitive function during the COVID-19 pandemic and adjusts for sufficient variables is needed. This study aimed to investigate the impact of the COVID-19 pandemic on cognitive function in the elderly by analyzing the database of the Korean Frailty and Aging Cohort Study (KFACS).

## 2. Materials and Methods

### 2.1. Study Population

The KFACS is a nationwide multicenter longitudinal study with the baseline survey conducted in 2016 and 2017, to identify the factors that contribute to frailty and aging in community-dwelling individuals aged 70–84 years [15]. The baseline survey for the cohort study was conducted at 10 centers, with 1559 participants in 2016 and 1455 participants in 2017 [16]. Follow-up was conducted at two-year intervals. Participants were recruited from urban, suburban, and rural communities nationwide. To determine the prevalence of frailty among three age groups (70–74, 75–79, and 80–84 years) and to consider the higher attrition rate in the oldest age group, the KFACS cohort adopted quota sampling stratified by age (70–74, 75–79, and 80–84 years, respectively, with a ratio of 6:5:4). This quota sampling is based on oversampling of the older group using population distribution data from the Korean Population and Housing Census conducted by Statistics Korea in 2015 (distributions of 43.5%, 33.8%, and 22.7% for adults aged 70–74 years, 75–79 years, and 80–84 years, respectively) [17]. Participants were recruited from diverse settings (local senior welfare centers, community health centers, apartments, housing complexes, and outpatient clinics) to minimize the selection bias. All participants were ambulatory, with or without the use of walking aids.

### 2.2. Intergroup Comparison over 2-Year Follow-Up

**Figure 1:**
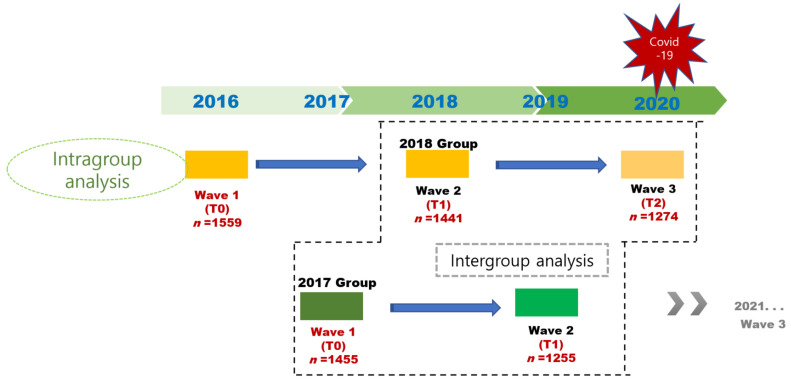
KFACS cohort scheme and intergroup analysis, intragroup analysis.

**Figure 2:**
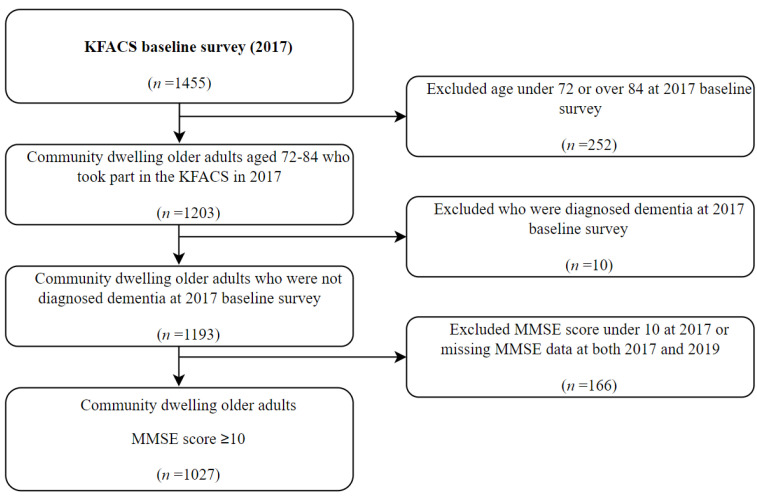
Flowchart of the study population of KFACS 2017 group.

**Figure 3:**
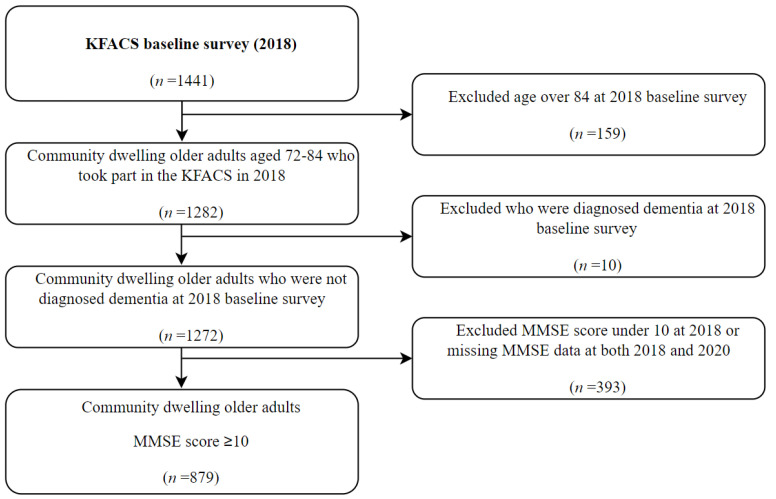
Flowchart of the study population of KFACS 2018 group.

We selected participants from the database of the 2017 and 2018 survey for intergroup comparison over 2-year follow-up (Figure 1). Data on the year 2020 of the 2018 group were evaluated for the COVID-19 pandemic. The participants included in the analysis were aged 72 to 84 years to equalize the age distribution of the 2017 and 2018 groups, as the age of the cohort in 2016 and 2017 at baseline was 70–84 years, and the age of the cohort in 2018 and 2019 at 2-year follow-up was 72–86 years. As shown in Figure 2, the 2017 group included 1027 participants, excluding those under 72 years or over 84 years in 2017 (*n* = 252) and those who were diagnosed with dementia in 2017 (*n* = 10) or with MMSE score < 10 in 2017 or missing MMSE data in both 2017 and 2019 (*n* = 166). As shown in Figure 3, the 2018 group included 879 participants, excluding those over 84 years in 2018 (*n* = 159) and those who were diagnosed with dementia in 2018 (*n* = 10) or with MMSE score <10 in 2018 or missing MMSE data in both 2018 and 2020 (*n* = 393).

### 2.3. Intragroup Comparison of 2018-Group

To examine the decline in cognitive function over two-year follow-ups from 2016 to 2018 and from 2018 to 2020, all available data from the 2018 group on 2016, 2018, and 2020 were used for the analysis (Figure 1). Again, data on 2020 from the 2018 group were evaluated for the COVID-19 pandemic.

The KFACS protocol was approved by the Institutional Review Board (IRB) of the Clinical Research Ethics Committee of Kyung Hee University Medical Center (IRB number: 2015-12-103), and all participants provided written informed consent. The data were anonymized to protect participants’ privacy. This study was conducted in accordance with the consensus ethical principles derived from the guidelines, including the Declaration of Helsinki.

### 2.4. Assessment of Cognitive Function

The KFACS carried out neuropsychological tests (CERAD-K, the Consortium to Establish a Registry for Alzheimer’s Disease Assessment Battery) and the Korean version of the Frontal Assessment Battery to appraise comprehensive cognitive function. The CERAD-K is a standardized clinical and neuropsychological assessment battery for the evaluation of patients with Alzheimer’s disease. The CERAD-K initially consisted of eight tests (Verbal Fluency, Modified Boston Naming, Mini-Mental State Examination (MMSE-KC), Word List Memory, Constructional Praxis, Word List Recall, Word List Recognition, and Constructional Praxis Recall). However, Word List Memory/Recall/Recognition, 7-Digit Span (forward, backward), Trail Making Test A(TMT-A), and MMSE-KC are included in this study [18].

The Word List Memory is a test that assesses memory for new information learning. It involves presenting 10 commonly used words at intervals of 2 s (seconds) and reading the words, followed by immediate recall of as many words as possible for 90 s. The total score is 30 points with 10 points per session. The Word List Recall test evaluates the ability for delayed recall of the given 10 words from the Word List Memory task. A maximum of 90 s is allowed, and the maximum score is 10. The Word List Recognition test measures recognition ability. The target is to distinguish between the 10 words presented in the Word List Memory test and 10 new words. The maximum score is 10. TMT A assesses attention, ordering, executive function, time-space search, and mental motion velocity. Patients were asked to draw a line connecting the numbers from 1 to 25 in ascending order, and the time (seconds) was recorded. Participants who did not complete the task over 360 s were excluded. The 7-Digit Span test assesses short-term and working memory. The participants hear seven digits of the number and then verbally repeat the sequence, as forward or backward. Digit span forward and backward was composed of 7-digit questions and repeated twice. Participants score one point when each digit was correctly recalled, and the total score was 14 points for each digit span forward and backward. The digit span total is the combined score of the digit span forward and backward.

The Frontal Assessment Battery (FAB) is a test that assesses executive functions such as planning, working memory, mental flexibility, and inhibition. It consists of similarities (conceptualization), lexical verbal fluency (mental flexibility), motor series (programming), conflicting instructions (sensitivity to interference), Go–No Go (inhibitory control), and prehension behavior (environmental autonomy), with a total score of 18; higher scores indicate better frontal lobe function [16].

### 2.5. Other Measurements

Demographic information such as study period, age, sex, independent living, marital status, living area, and years of education were investigated through face-to-face interviews every year. Social characteristics, such as occupation, social activity, and household income were also investigated. Social activity was defined as having at least one meeting or group activity with friends or colleagues. Physical activity levels were divided into two groups in this study: physical activity less than three times a week was defined as low physical activity, and that more than three times a week was defined as high physical activity. Examples of physical activities surveyed in the questionnaire included walking fast (at work), carrying light objects, cleaning (outdoors), parenting (bathing, hugging, etc.), lifting or carrying heavy things (about 20 kg or more), digging, labor at construction sites, and carrying by stairs. Clinical conditions such as hypertension, DM, dyslipidemia, major depressive disorder, dementia, cerebrovascular disease, malignancy, and BMI were also assessed. Vitamin D deficiency (<10 ng/mL) or insufficiency (10–30 ng/mL) was assessed based on laboratory findings.

### 2.6. Statistical Analysis

Data are presented as mean ± standard deviation (SD) or as percentages. Continuous variables were compared using independent and paired t tests. Categorical variables were compared using the chi-squared test. A multiple generalized linear model was used to adjust for confounding variables in the intergroup comparison. Generalized linear models were used to adjust for confounding variables (age, sex, marital status, living alone, area, education year, occupation, social activity, sleeping time, dyslipidemia, baseline physical activity, physical activity after 2 years, depression after 2 years, and the period of study). Additionally, the paired t tests and multiple mixed models were used for intragroup comparisons. All statistical analyses were performed using IBM SPSS Statistics for Windows (version 25. Armonk, NY, USA). Statistical significance was set at *p* < 0.05.

## 3. Results

### 3.1. General Characteristics of the Study Population

The mean age of the 2017 group (77.0 ± 3.1 years) was slightly lower than that of the 2018 group (77.6 ± 3.3 years). In the 2017 group, 214 participants (20.8%) lived alone, while 228 (25.9%) lived alone in the 2018 group. Mean education period of the 2017 group was 9.1 ± 5.0 years and that of the 2018 group was 8.1 ± 4.9 years (*p* < 0.0001). Regarding physical activity, 464 (45.2%) and 445 (50.6%) participants reported low physical activity in the 2017 and 2018 groups, respectively (*p* = 0.0177) (Table 1).

### 3.2. Two-Year Decline of Cognitive Functions in the 2017 and 2018 Groups (Intergroup Comparison)

As shown in Table 2, we evaluated 2-year differences in cognitive functions in the 2017 and 2018 groups. The Word List Memory score decreased by −0.14 from 2018 to 2020, while the score increased from 2017 to 2019 by 0.53 (*p* < 0.0001). The Word List Recall score decreased from 2018 to 2020 with a −0.25 difference, while the score increased from 2017 to 2019 with a 0.03 difference (*p* = 0.0002).

Confounding variables for intergroup comparisons were selected from the statistically significant variables in Table 1. The confounding variables were age, sex, marital status, living alone, area, years of education, occupation, social activity, sleeping time, dyslipidemia, baseline physical activity, physical activity after 2 years, depression after 2 years, and the period taken for the next follow-up, and they were adjusted in logistic regression models (Table 3). Model 1 was an unadjusted intergroup comparison of differences in cognitive function. Model 2 was adjusted for age and gender. Model 3 was adjusted for Model 2 plus marital status, living alone, residential area, years of education, occupation, social activity, sleeping time, dyslipidemia, and baseline physical activity. Model 4 was adjusted in the same manner as Model 3 plus physical activity after 2 years and depression after 2 years. Finally, Model 5 was adjusted to be the same as Model 4 plus the study period until the next survey (Table 3). In the unadjusted logistic regression model (Model 1), word list memory and word list recall scores decreased more in the 2018 group than in the 2017 group (Table 3). The word list memory score decreased from 2018 to 2020 with −0.14 difference, while the score increased from 2017 to 2019 with a 0.53 difference (*p* < 0.0001). The word list recall score decreased from 2018 to 2020 with a −0.245 difference, while the score increased from 2017 to 2019 with a 0.03 difference (*p* = 0.0006). In Model 5, the word list memory score increased from 2018 to 2020 with a difference of 0.28, while the score increased from 2017 to 2019 with a difference of 0.84 (*p* = 0.0017). The word list recall score increased from 2018 to 2020 with a difference of 0.22, while the score increased from 2017 to 2019 with 0.45 difference (*p* = 0.0085).

### 3.3. Changes in Cognitive Functions from 2016 to 2020 of 2018 Group (Intragroup Comparison)

In Table 4, we evaluated the mean differences in cognitive function test scores from 2016 to 2020 for intragroup comparisons. The mean difference in the word list memory score from 2018 to 2020 was −0.14, whereas that from 2016 to 2018 was 0.47, which was a significant difference (*p* = 0.0028). The mean difference in the word list recall score from 2018 to 2020 was −0.25, while that from 2016 to 2018 was 0.02, which was significant (*p* = 0.0057). The mean difference of the 7-digit span score from 2018 to 2020 was −0.26, while that from 2016 to 2018 was −1.19 (*p*-value < 0.0001). The mean difference of the FAB score from 2018 to 2020 was 0.23, while that from 2016 to 2018 was −0.34 (*p*-value 0.0001). Thus, the mean differences of the 7-digit span score from 2018 to 2020 decreased much lower than that from 2016 to 2018. The mean differences in FAB scores from 2018 to 2020 showed a tendency to increase more than those from 2016 to 2018. These scores were statistically significant, as well as in the multiple mixed model (Table 4).

## 4. Discussion

The decline in the cognitive function of the elderly Koreans included in this study was more during the COVID-19 pandemic than during the pre-pandemic period, especially with regards to memory and recall.

The COVID-19 pandemic has undoubtedly had a greater impact on patients who are already suffering from cognitive impairment or have already been diagnosed with dementia [19]. One study that included participants with dementia found a significant decrease in MMSE points per year for the year 2020 (the year the COVID-19 pandemic started) compared with other years [20]. Another study suggested that the cognitive function of patients with dementia or mild cognitive impairment (MCI) declined more rapidly during the COVID-19 lockdown than during the pre-pandemic period [8].

However, studies showing accelerated cognitive decline in community-dwelling older adults without dementia during the COVID-19 pandemic are rare [13,14]. Moreover, the above-mentioned studies used subjective questionnaires or telephone interviews for cognitive function tests, and there were no comparative groups. In this study, we ascertained the effect of the COVID-19 pandemic on cognitive decline by making comparison with a control cohort unaffected by the COVID-19 pandemic. We found that word memory and recall were significantly affected as shown by the cognitive function tests.

There may be several reasons for the accelerated cognitive decline in community-dwelling older adults during the COVID-19 pandemic. Higher physical activity levels have been shown to be associated with better cognitive function in older adults [21]. Therefore, a reduction in physical activity due to social distancing may be associated with subjective memory decline in adults [22]. Decreased social activities or social isolation during the COVID-19 pandemic may be another cause of the accelerated cognitive decline. This finding is similar to that of a recent study in which loneliness was shown to be associated with cognition among community-dwelling older adults [23]. Research has also shown that perceived social isolation (i.e., loneliness) is a risk factor for increased cognitive decline [24]. In fact, social isolation during the COVID-19 pandemic has been reported to be associated with a decline in subjective cognitive function among older adults in a Japanese cohort [13]. Additionally, because social isolation is associated with an increased risk of depression, depression may aggravate cognitive decline during the COVID-19 pandemic. Compared to the pre-pandemic period, higher incidence of depression was reported during the lockdown, particularly in those aged ≥70 years and/or living alone [25].

However, as seen in Table 2, the accelerated decrease in word list memory and word list recall function during the COVID-19 pandemic was still significant compared to the control cohort, even after adjusting for physical activities, social activities, and depression. Other reasons for this observation are needed. One such reason is that social isolation during the COVID-19 pandemic has been shown to be associated with chronic inflammation [26]. Chronic systemic inflammation has been postulated to be a risk factor for neurodegenerative disorders and cognitive decline [27]. Another probable reason is the fear of the COVID-19 infection. One study postulated that the fear of the COVID-19 contagion can cause cognitive impairments, especially in memory and learning [28].

Meanwhile, in the intragroup comparison in this study, digit span and FAB score tended to decrease in 2020 during the COVID-19 pandemic. Authors wanted to show the trajectory of changes in cognitive functions in the same cohort (intragroup) as a back-up evidence of the intergroup comparison. The intragroup comparison did not involve the control group, therefore the confounding factors were not controlled. Therefore, this result could be the possibility of a random error.

The current study had some limitations. First, because this study only included community-dwelling older adults, it is difficult to generalize the results of the study to older adults who are hospitalized or bedridden in nursing homes. Therefore, the results of the study may not be generalizable to other populations, and may be inapplicable to other settings. Second, we excluded individuals whose MMSE score was <10 or those who were previously diagnosed with dementia; however, some participants with mild dementia may have been included. Third, more biologic factors should have been added for analysis of reasons for accelerated cognitive decline during the COVID-19 pandemic. The inflammatory marker of CRP (C-Reactive Protein) may not account for accelerated cognitive decline during the COVID-19 pandemic. Previously, we evaluated 2-year differences in CRP (C-Reactive Protein) levels in the 2017 and 2018 groups, but the difference was insignificant. Finally, we did not investigate whether the participants had caught COVID-19. However, referring to a report from the residual serum samples of participants recruited between 24 April and 12 December 2020 from Korea National Health and Nutrition Examination Survey (KNHANES) including 19.0% of 70 or older, the seropositivity rate was just 0.09% (5/5284). It means there were few hidden undiagnosed infections (asymptomatic patients) in the community in 2020, due to the thorough 3T strategy (testing, tracing, treatment) [29]. Therefore, we guess that the participants in this study, who were community-dwelling older adults, had much lower rate of having caught COVID-19. Still, a few of the participants could have caught COVID-19, and it could affect the association of cognitive decline with the COVID-19 pandemic. Nevertheless, a key strength of this study is that we used a large nationwide multicenter cohort sample including community-dwelling older Korean adults.

## 5. Conclusions

The cognitive function of the Korean elderly cohort declined much more during the COVID-19 pandemic than the pre-pandemic cohort, especially in terms of memory and recall function. Additionally, accelerated cognitive decline was prominent even after adjusting for physical activity, social activity, and depression.

## Figures and Tables

**Table 1:** Baseline characteristics of the study subjects according to intergroup comparison.

Variables	2017-Group (*n* = 1027)	2018-Group (*n* = 879)	*p* Value
**Demographics**			
Age	77.0 ± 3.1	77.6 ± 3.3	0.0004 *
Male sex	500(48.7%)	415(47.2%)	0.5212
Married	708(68.9%)	559(63.7%)	0.0151
Living alone	214(20.8%)	228(25.9%)	0.0085
Urban living	254(24.9%)	277(31.6%)	0.0012
Education years	9.1 ± 5.0	8.1 ± 4.9	<0.0001 *
Body mass index			
BMI < 19	29(2.8%)	25(2.8%)	0.3902
BMI ≥ 23	714(69.5%)	586(66.7%)	
**Social Characteristics**			
Occupation	283(27.6%)	286(32.5%)	0.0185
Social activity	964(93.9%)	831(94.5%)	0.5313
House hold income			
Low	75(7.3%)	51(5.8%)	0.1871
High	950(92.7%)	827(94.2%)	
**Health behavior**			
Current smoker	389(38.0%)	340(38.7%)	0.7420
Heavy Alcohol drinking	179(24.6%)	132(20.9%)	0.1080
Low physical activity at baseline	464(45.2%)	445(50.6%)	0.0177
Low physical activity after 2 years	497(48.4%)	507(57.7%)	<0.0001
**Clinical conditions**			
Hypertension	622(60.6%)	526(60.0%)	0.7738
Dyslipidemia	348(34.2%)	360(41.8%)	0.0007
Diabetes mellitus	244(23.8%)	205(23.3%)	0.8137
Cerebrovascular disease	51(5%)	42(4.8%)	0.8502
Cancer	29(2.8%)	27(3.1%)	0.7494
Vitamin d deficiency or insufficiency	766(74.6%)	641(73.0%)	0.4342
Depression at baseline	222(21.6%)	188(21.4%)	0.9037
Depression after 2 years	257(25.0%)	257(29.2%)	0.0388
**Period until the next survey (day)**	702 ± 51.7	743.1 ± 43.1	<0.0001 *

Notes: All values are presented as the mean ± standard deviation or number (%). Statistical significance was set at *p* < 0.05. *p*-values were based on the chi-squared test and independent *t* test (*); urban living and education years of 2018 data were replaced with 2016 data (no data for 2018); BMI normal range: 19 ≤ BMI < 23, social activity was defined as having at least one meeting or group activity with friends or colleagues (Yes or No); heavy alcohol drinking implies two or more weekly alcohol drinks (once seven cups for male, five cups for female).

**Table 2:** Unadjusted mean differences of cognitive function during 2 years in 2017-Group and 2018-Group (intergroup comparison).

	2017-Group									2018-Group									Cohen’s d	*p*-Value *
	2017 Year			2019 Year			Mean Diff. (2019–2017)			2018 Year			2020 Year			Mean Diff. (2020–2018)				
	*n*	Mean	SD	*n*	Mean	SD	*n*	Mean	SD	*n*	Mean	SD	*n*	Mean	SD	*n*	Mean	SD		
MMSE	1027	25.67	3.13	1027	25.39	3.31	1027	−0.28	2.57	879	25.59	3.19	879	25.23	3.59	879	−0.36	2.34	−0.0334	0.4640
Word List Memory	1027	16.66	4.26	1025	17.20	4.49	1025	0.53	3.25	874	17.74	4.40	877	17.59	4.56	873	−0.14	3.56	−0.1963	<0.0001
Word List Recall	1027	5.51	2.08	1024	5.55	2.21	1024	0.03	1.64	873	5.81	2.10	877	5.56	2.24	872	−0.25	1.67	−0.1712	0.0002
Word List Recognition	1026	8.64	1.73	1026	8.62	1.83	1025	−0.01	1.60	873	8.70	1.75	877	8.57	1.95	872	−0.13	1.59	−0.0773	0.0937
TMT	1014	76.30	51.44	1004	76.67	51.54	1001	1.64	34.13	848	81.75	54.09	849	82.53	54.48	832	1.33	33.78	−0.0089	0.8501
7-Digit span	1026	10.47	3.72	1026	10.12	3.68	1025	−0.35	2.58	876	9.54	3.57	877	9.29	3.59	875	−0.26	2.38	0.0367	0.4218
FAB	1026	13.65	2.90	1027	13.67	3.01	1026	0.02	2.49	876	13.10	3.14	877	13.33	3.23	875	0.23	2.43	0.0852	0.0644

Statistical significance was set at *p* < 0.05. *p*-values were based on the independent t test for the difference (*); the effect size of Cohen’s d for two groups is (Mean diff. (2020–2018)—Mean diff. (2019–2017))/SD_pooled_; MMSE: mini-mental state examination; recall test (total score of 10); recognition test (total score of 10); TMT (Trail-Making Test, out of 360 s, increasingly worse); Span (Digit span test, total score of 28); FAB: Frontal Assessment Battery (total score of 18).

**Table 3:** Estimated mean differences of cognitive function during 2 years in 2017-Group and 2018-Group (intergroup comparison).

Dependent Variable (Difference)	Group	Model 1 (Unadjusted)				Model 2				Model 3				Model 4				Model 5			
		Mean Diff. *	CI		*p*-Value	Mean Diff. *	CI		*p*-Value	Mean Diff. *	CI		*p*-Value	Mean Diff. *	CI		*p*-Value	Mean Diff. *	CI		*p*-Value
MMSE	2017	−0.28	−0.43	−0.13	0.4672	−0.28	−0.43	−0.13	0.5243	−0.16	−1.11	0.79	0.5623	−0.21	−1.17	0.74	0.5271	−0.22	−1.18	0.73	0.6680
	2018	−0.36	−0.52	−0.20		−0.35	−0.52	−0.19		−0.23	−1.18	0.72		−0.29	−1.24	0.67		−0.28	−1.24	0.68	
Word List Memory	2017	0.53	0.32	0.74	<0.0001	0.52	0.31	0.72	<0.0001	0.99	−0.32	2.30	0.0002	0.85	−0.46	2.17	0.0005	0.84	−0.48	2.16	0.0017
	2018	−0.14	−0.36	0.09		−0.12	−0.35	0.10		0.39	−0.92	1.70		0.28	−1.03	1.59		0.28	−1.03	1.60	
Word List Recall	2017	0.03	−0.07	0.14	0.0002	0.02	−0.08	0.13	0.0006	0.49	−0.14	1.13	0.0023	0.46	−0.18	1.09	0.0031	0.45	−0.19	1.09	0.0085
	2018	−0.25	−0.36	−0.14		−0.24	−0.35	−0.13		0.25	−0.38	0.89		0.22	−0.42	0.86		0.22	−0.42	0.86	
Word List Recognition	2017	−0.01	−0.11	0.09	0.0937	−0.02	−0.12	0.08	0.1930	0.26	−0.36	0.87	0.1263	0.23	−0.39	0.85	0.1385	0.23	−0.39	0.85	0.1303
	2018	−0.13	−0.24	−0.03		−0.12	−0.22	−0.01		0.14	−0.48	0.75		0.12	−0.50	0.73		0.11	−0.51	0.73	
TMT	2017	1.64	−0.47	3.74	0.8501	1.71	−0.40	3.82	0.7790	−9.23	−22.35	3.89	0.9899	−7.98	−21.17	5.20	0.9446	−7.67	−20.88	5.55	0.7681
	2018	1.33	−0.98	3.64		1.26	−1.05	3.58		−9.25	−22.36	3.86		−8.10	−21.27	5.08		−8.20	−21.39	4.99	
7-Digit Span	2017	−0.35	−0.51	−0.20	0.4248	−0.36	−0.52	−0.21	0.3468	0.32	−0.64	1.27	0.5530	0.26	−0.70	1.23	0.5658	0.25	−0.72	1.21	0.4238
	2018	−0.26	−0.43	−0.10		−0.26	−0.42	−0.09		0.39	−0.57	1.34		0.33	−0.63	1.29		0.35	−0.61	1.31	
FAB	2017	0.02	−0.13	0.17	0.0644	0.02	−0.13	0.17	0.0585	−0.06	−1.00	0.89	0.0699	−0.06	−1.01	0.90	0.0574	−0.03	−0.99	0.92	0.1164
	2018	0.23	0.07	0.40		0.24	0.07	0.40		0.16	−0.79	1.10		0.17	−0.78	1.12		0.17	−0.79	1.12	

Notes: statistical significance was set at *p* < 0.05. *p*-values were based on a multiple generalized linear model. Mean Diff. *: Estimated mean value by least squares; CI: confidence interval; MMSE: mini-mental state examination; recall test (total score of 10); recognition test (total score of 10); TMT (Trail-Making Test, out of 360 s, increasingly worse); Span (Digit span test, total score of 28); FAB: Frontal Assessment Battery (total score of 18). Model 1: Unadjusted intergroup comparison of cognitive function mean difference; Model 2: Intergroup comparison of cognitive function mean difference adjusted by age and sex; Model 3: Adjusted as in Model 2 plus marital status, living alone, area, education years, occupation, social activity, sleeping time, dyslipidemia, and baseline physical activity; Model 4: Adjusted as in Model 3 plus physical activity after 2 years and depression after 2 years; Model 5: Adjusted as in Model 5 plus study period.

**Table 4:** Mean differences of cognitive function according to intragroup comparison.

	Mean Difference (2018–2016)			Mean Difference (2020–2018)			Intragroup Mean Difference Comparison (2020–2018)-(2018–2016)			Cohen’s d	*p* Value *	*p* Value **
	*n*	Mean	SD	*n*	Mean	SD	*n*	Mean	SD			
MMSE	879	–0.15	2.73	879	−0.36	2.34	879	−0.21	4.10	−0.05	0.1301	0.0841
Word List Memory	873	0.47	3.53	873	−0.14	3.56	873	−0.61	6.03	−0.10	0.0028	0.0003
Word List Recall	872	0.02	1.71	872	−0.25	1.67	872	−0.27	2.85	−0.09	0.0057	0.0010
Word List Recognition	872	−0.02	1.63	872	−0.13	1.59	872	−0.12	2.67	−0.04	0.1997	0.1327
TMT	827	2.77	34.47	827	1.00	33.42	827	−1.77	56.51	−0.03	0.3677	0.2889
7-digit Span	875	−1.19	2.73	875	−0.26	2.38	875	0.93	4.30	0.22	<0.0001	<0.0001
FAB	875	−0.34	2.70	875	0.23	2.43	875	0.57	4.43	0.13	0.0001	<0.0001

Notes: Statistical significance was set at *p* < 0.05 (statistically significant). The effect size of Cohen’s d for two paired groups is (Intragroup mean difference comparison—0)/SD. * *p*-values are based on the paired *t* test. ** *p*-values are based on the multiple mixed model adjusted age, sex for repeated data; MMSE (Mini-Mental State Examination); recall test (total score of 10); recognition test (total score of 10); TMT (Trail-Making Test, out of 360 s, increasingly worse); Span (digit span test, total score of 28); FAB (Frontal Assessment Battery, total score of 18).

## Data Availability

The data presented in this study are available from the authors upon reasonable request.
